# Examining the Relationship Between Sarcopenia and Rotator Cuff Tears: A Retrospective Comparative Study

**DOI:** 10.3390/jcm14010220

**Published:** 2025-01-02

**Authors:** Jae-Hwa Kim, Inseok Jang, Simho Jeong, Jeeseop Shin, Siyeong Yoon, Hyunil Lee, Soonchul Lee

**Affiliations:** 1 Department of Orthopaedic Surgery, CHA Bundang Medical Center, CHA University School of Medicine, 335 Pangyo-ro, Bundang-gu, Seongnam-si 13488, Gyeonggi-do, Republic of Korea; drjkim@cha.ac.kr (J.-H.K.); isjamg21@naver.com (I.J.); a226050@chamc.co.kr (J.S.); tldud1105@naver.com (S.Y.); 2 Department of Orthopedic Surgery, Ilsan Paik Hospital, Inje University, 170, Juhwa-ro, Ilsangeo-gu, Goyang-si 10380, Gyeonggi-do, Republic of Korea; lhi76@naver.com

**Keywords:** sarcopenia, rotator cuff tear, body composition test

## Abstract

**Background**: With the rapid increase in the aging population, the number of patients complaining of shoulder pain is also increasing. Among shoulder ailments, rotator cuff tears (RCTs) are most frequently observed in middle-aged and elderly individuals. Sarcopenia refers to the decline in muscle mass (lean body mass) and the subsequent decrease in muscle function that is linked to the natural aging process. To the best of our knowledge, there is currently limited information available regarding the association between RCTs and sarcopenia. **Methods:** The study included only individuals who had undergone dual-energy X-ray absorptiometry and body composition assessments. After applying the exclusion criteria, the participants were divided into sarcopenia and non-sarcopenia groups by the body composition tests. Next, those diagnosed with RCTs were assigned to the experimental group, and those without RCTs were assigned to the control group. The matching was performed using propensity score matching. Sarcopenia was defined as a skeletal muscle index lower than 7.0 kg/m^2^ in males and 5.4 kg/m^2^ in females. Multivariable logistic regression with backward elimination was performed. **Results:** After propensity score matching, there were no significant differences in age, sex, and bone mineral density between the RCT and control groups. In the univariate analysis, it was observed that most of the baseline data and demographic factors did not exhibit significant differences, except for calcium levels and the presence of chronic kidney injury. According to the multivariable logistic regression analysis of factors related to RCTs, sarcopenia was unrelated to RCTs, but chronic kidney injury and Ca levels were significantly associated. Also, there was also no significant association between sarcopenia and RCT size and severity. **Conclusions:** In conclusion, we cannot find a significant relationship between sarcopenia and RCTs.

## 1. Introduction

As the global demographic of older adults continues to grow, there has been a notable surge in reports of shoulder pain, which has consequently sparked increased interest and concern regarding shoulder conditions [1]. This heightened focus extends beyond not only daily life activities and occupational tasks, but also to the realm of sports and physical exercise. Within this spectrum of shoulder disorders, rotator cuff tear (RCT) emerges as the most prevalent condition affecting middle-aged and elderly individuals. These tears represent the primary cause necessitating shoulder surgeries among these age groups [2]. As such, RCTs are the most common culprits behind shoulder disabilities, leading to significant functional impairments, particularly in adults aged 50 years and older [3,4].

The etiology of RCTs is multifaceted, involving a complex interplay of age-associated degenerative changes alongside the influences of microtrauma and macrotrauma [5]. Such wear and tear over time contributes to the weakening and subsequent tearing of the rotator cuff tendons Furthermore, diseases, including diabetes mellitus, hypercholesterolemia, thyroid dysfunction, and osteoporosis, have been identified as influential factors in the development and progression of RCTs [6]. The chronic nature of these tears often results in diminished shoulder function and muscle atrophy, presenting further complications in affected individuals.

The term “sarcopenia” was introduced by Rosenberg in 1989 to describe the reduction of muscle mass, or lean body mass, and the consequent decline in muscle functionality because of the aging process. Today, sarcopenia is recognized as a critical clinical issue impacting the elderly population, with the field of research concerning this condition rapidly expanding. The prevalence of sarcopenia is reported to range from 5% to 13% in individuals between the ages of 60 and 70, while this percentage escalates to between 11% and 50% in those aged 80 and above [7]. Recent studies have illuminated a strong association between sarcopenia and a variety of diseases, including obesity, diabetes mellitus, hypercholesterolemia, cardiovascular disease, osteoporosis, and fractures [8,9]. In terms of prognosis, sarcopenia is closely linked with increased physical disability, prolonged hospital stays, greater postoperative complications, and heightened mortality rates [10,11,12,13].

Given the age-related increase in the prevalence of RCTs, a significant association between RCT and sarcopenia seems plausible [14]. Nevertheless, our current knowledge reveals a scarcity of comprehensive data on this specific relationship. The limited research available presents conflicting findings, with some studies suggesting that individuals with sarcopenia might have a reduced likelihood of encountering severe rotator cuff complications such as tendon ruptures. These varied and at times contradictory outcomes have led us to pursue more detailed investigations into this area [6,15,16,17].

In this study, we postulated that the prevalence of sarcopenia would be disproportionally higher among patients diagnosed with RCTs compared to those in a control group without RCTs. Additionally, we speculated that the severity of sarcopenia would be directly related to the size of the RCT. Thus, our objective was to determine whether there is a notable difference in the prevalence of sarcopenia between RCT-affected and non-RCT individuals, and to uncover any potential relationship between sarcopenia and RCT size and severity, employing propensity score matching (PSM) as a precise analytical tool. By elucidating this association, we aim to enhance the comprehensive management approaches for patients undergoing treatment for RCTs, potentially improving their overall clinical outcomes.

## 2. Materials and Methods

### 2.1. Study Design and Study Population

This was a retrospective comparative study of patients who visited our institution between January 2010 and December 2020. The study protocol was reviewed and approved by the Institutional Review Board of the CHA Bundang medical center, Korea (No. 2024-10-033). The requirement for written informed consent was waived because this was a retrospective study. Adults aged ≥40 years who underwent both dual-energy X-ray absorptiometry (DEXA) and body composition tests at our institution were reviewed. Patients aged >85 years (n = 4), patients with severe comorbidities that influenced musculoskeletal activities (e.g., cerebrovascular accident, bedridden status, cancer and so on) (n = 10), osteoporotic fractures, including spine or hip fractures (n = 28), and patients with high-energy trauma (n = 10) were excluded.

**Figure 1:**
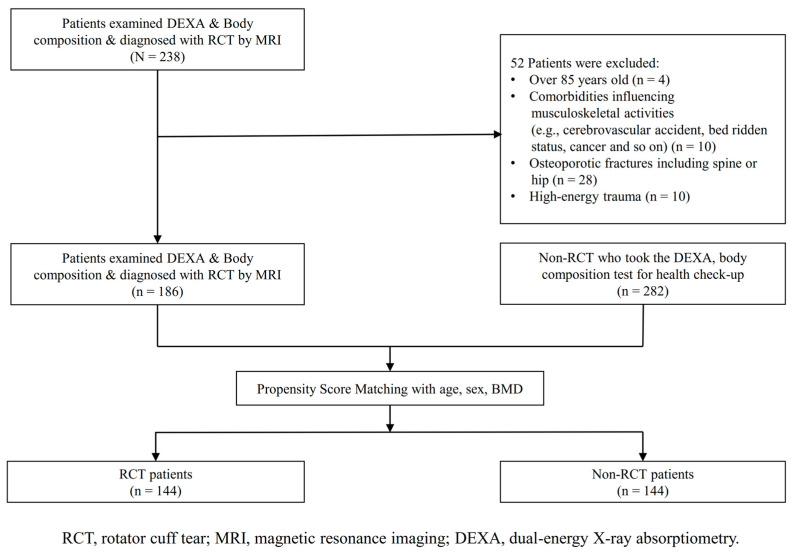
Study population flow chart for patient inclusion.

Among the patients who underwent DEXA and body composition tests, patients diagnosed with RCTs were included in the experimental group. Diagnosis was made based on both physical examination and magnetic resonance imaging (MRI) of the shoulder in all RCT patients. The non-RCT control group was mainly recruited from patients who visited the health promotion center of this institute, and we conducted a thorough past medical history survey on patients. These participants also did not have a history of severe comorbidities that influenced musculoskeletal injuries, osteoporotic fractures, and high-energy trauma. Also, they had no history of shoulder pain or known shoulder disease, and no history of shoulder surgery. The initial analyses included 186 patients in the experimental group and 282 in the control group. After data collection, we established an experimental group and a control group after matching data regarding age, sex, and bone mineral density (BMD). The matching was performed using PSM. To ensure there was no difference in the matching variables between the two groups, standardized mean differences were set as <±0.1. After this process, the RCT and control groups comprised 144 patients each (Figure 1).

### 2.2. Diagnostic Criteria for Sarcopenia

Because the study was retrospective, it relied on previously collected data. These measurements were used to analyze body composition results and to identify and categorize participants into sarcopenia groups. The appendicular skeletal muscle mass (ASM), which is the total muscle mass of all four limbs, was used to compute the skeletal muscle index (SMI). The SMI is calculated by dividing the ASM by the square of the height (SMI unit: kg/m^2^). To define sarcopenia, cut-off values from previous studies were applied as follows: an SMI lower than 7.0 kg/m^2^ for males and 5.4 kg/m^2^ for females [18]. The body mass index (BMI) was calculated by dividing body weight by the square of the height and was used as a measure of obesity in this study. Based on the results of the body composition tests, participants were classified into either the sarcopenia or non-sarcopenia groups [19].

### 2.3. Other Variables

Data on various demographics and health metrics such as age, sex, BMI, height, weight, body composition (measured by SMI), and BMD were collected. BMD was measured in the lumbar spine (L1–L4) and the proximal femur (femur neck and total hip) using dual-energy X-ray absorptiometry (DEXA) with the Lunar Prodigy Advance system from GE Lunar, located in Madison, WI, USA. According to the World Health Organization, osteopenia is diagnosed when the T-score standard deviation falls between −2.5 and −1.0, while osteoporosis is indicated by a T-score of −2.5 or lower [15,20]. Additionally, the participants’ medical histories, including conditions such as hypertension, diabetes mellitus, chronic kidney injury, and previous osteoporotic fractures, were thoroughly reviewed using electronic medical records. Laboratory tests were conducted to obtain detailed values for serum calcium (mg/dL), phosphorus (mg/dL), vitamin D (25(OH)D3) (ng/mL), and parathyroid hormone (PTH) (pg/mL). These samples were derived from venous blood and measured with specialized instruments. Serum calcium and phosphorus levels were determined using a Stat Profile^®^ Critical Care Xpress analyzer from Nova Biomedical, Waltham, MA, USA. Vitamin D levels were measured using a specific kit on the Cobra II Auto-γ Counting System from Packard Instruments, Downers Grove, IL, USA. Serum PTH levels were quantified using a standard enzyme-linked immunosorbent assay-PTH immunoradiometric assay from IBL International GmbH, Hamburg, Germany, which has a minimum detectable limit of 1.0 pg/mL. However, PTH levels were excluded from the final analysis because of a substantial amount of missing data.

### 2.4. Diagnosis of RCT

To assess the condition of the rotator cuff tendon, along with the size and severity of any tears, MRI data from all patients in the RCT group were analyzed. The MRI sequences included oblique coronal, oblique sagittal, and axial T2-weighted spin echo images. Based on these images, rotator cuff tears (RCTs) were classified as either full-thickness (FTRCTs) or partial-thickness tears (PTRCTs). FTRCTs were identified by a supraspinatus tendon defect spanning from the bursal to the articular surface. The shape of the tear was categorized using DeOrio and Cofield’s classifications: small (0–10 mm), medium (10–30 mm), large (30–50 mm), or massive (over 50 mm) in size [15]. PTRCT was defined by a defect in the supraspinatus tendon and further categorized into specific types such as articular-sided, bursal-sided, intra-tendinous, and combined type tears. Although MRI scans also showed tears in the subscapularis and infraspinatus tendons, these were not included due to inadequate data. PTRCTs were also classified based on both the location (A: articular, B: bursal, C: intra-tendinous) and the depth of the tear (Grade 1: less than 3 mm or 25% thickness, Grade 2: 3 to 6 mm or 25–50% thickness, Grade 3: more than 6 mm or over 50% thickness), according to Ellman et al. [21]. MRI characteristics of PTRCT included increased signal intensity and altered morphology without full-thickness discontinuity on T1 images, along with increased signal intensity and focal defect on T2 images. Differentiating PTRCT from tendinosis on MRI is key, as tendinosis typically shows high signal intensity on T1 and proton-density images but low intensity on T2 images [22]. In this study, the PTRCT group was incorporated into the small tear category of FTRCT. Three independent experts—one musculoskeletal radiologist and two senior orthopedic surgeons—reviewed the MRI images independently. In instances where their interpretations differed, the images were re-evaluated collectively, and the majority opinion was used for final decision-making.

### 2.5. Statistical Analysis

Data processing and statistical analysis were conducted using R software (version 3.6.3, The R Foundation for Statistical Computing, Vienna, Austria; accessed from http://www.r-project.org/ on 15 July 2022). For continuous data that followed a normal distribution, results were expressed as the mean plus or minus the standard deviation. Statistical significance was determined with a *p*-value threshold of less than 0.05. Comparing groups, Pearson’s chi-squared test was used for categorical data, and Student’s *t*-test was applied for continuous data. A univariate analysis was carried out to identify factors associated with RCTs. Subsequently, a multivariable logistic regression analysis was performed to account for and confirm the influence of various factors associated with RCTs. The backward elimination method was applied using specific *p*-values, where univariate variables with a *p*-value less than 0.20 were included in the model. For the multivariable logistic regression, continuous variables were categorized into two groups (high and low groups) based on the mean value. The results of these analyses are presented as odds ratios (ORs) with 95% confidence intervals (CIs). PSM was used to minimize potential bias due to differences in patient demographics. Initially, covariates like age, sex, and BMD were used to calculate the propensity scores. The RCT group was then matched to the non-RCT group using greedy nearest-neighbor matching in a fixed 1:1 ratio. Age was specifically selected as a covariate. The quality of the matching was evaluated by comparing standardized mean differences, ensuring equivalent numbers of participants between the RCT and non-RCT groups.

## 3. Results

### 3.1. Differences Between the Sarcopenia and Non-Sarcopenia Groups Before PSM

Among the 468 participants, 176 were identified as having sarcopenia, while 292 were classified in the non-sarcopenia group. Before PSM, there were significant differences between these two groups regarding age, sex, weight, BMI, BMD, calcium levels, and chronic kidney injury, with *p*-values of 0.003, 0.012, <0.001, <0.001, <0.001, 0.001, and <0.001, respectively. The sarcopenia group had significantly higher age, female proportion, and chronic kidney injury proportion, but lower weight, BMI, BMD, and calcium levels compared to the non-sarcopenia group. However, the differences in the other variables were not significant.

**Figure 2:**
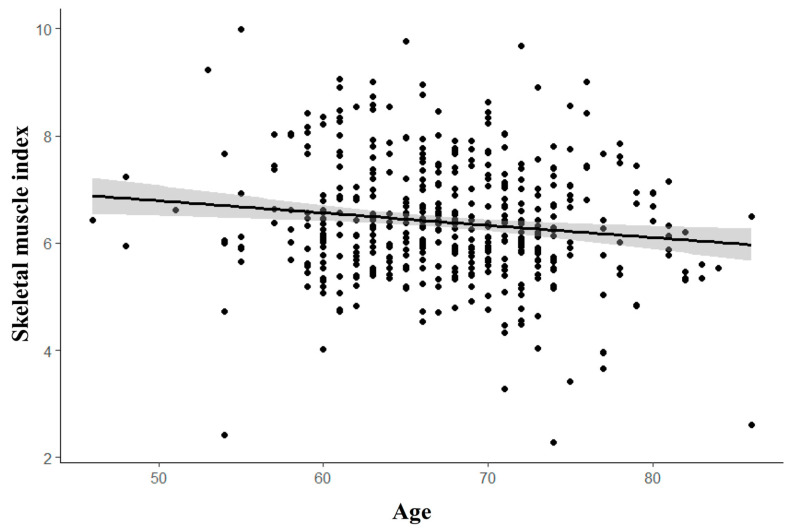
Correlation of SMI (skeletal muscle index) and age. The correlation between age and skeletal muscle index (SMI) is illustrated by a trend line, indicating that as individuals age, the SMI decreases.

However, no association was found between the presence of sarcopenia and RCT, with a *p*-value of 0.441, as detailed in Table 1. Regardless of sarcopenia status, skeletal muscle mass tends to decrease with age (R^2^ = 0.018, *p* = 0.003), as illustrated in Figure 2.

### 3.2. Demographic Characteristics Between Non-RCT and RCT Groups After PSM

After PSM, the standardized mean differences between the non-RCT and RCT groups were 0.01 for age, −0.02 for sex, and 0.03 for BMD, as shown in Supplementary Figure S1. Both groups had an average age of 68 years and a BMD T-score of −1.5. In the univariate analysis comparing the RCT and non-RCT control groups, there were no significant differences in most baseline and demographic factors, including underlying conditions (such as hypertension, diabetes mellitus, chronic kidney injury, and osteoporotic fractures), BMI, BMD, and overall body status (all *p*-values greater than 0.05). However, significant differences were found for calcium (*p* = 0.028) and chronic kidney injury (*p* = 0.005), as presented in Table 2.

### 3.3. Relationship Between Sarcopenia and RCT Sizes or Severity

Among the 144 patients with RCT, 66 patients had small tears, 34 had medium tears, 24 had large tears, and 20 had massive tears. There were significant differences in age (*p* = 0.041) and sex (*p* = 0.049) across these groups. As age increased, there was a tendency for RCT size to increase. However, no significant association was found between sarcopenia and RCT size (*p* = 0.183), as shown in Table 3. Regarding RCT severity, 40 cases were identified as PTRCT and 104 as FTRCT. A significant age difference was observed between these groups, with a *p*-value of 0.002; the average age for the PTRCT group was 65 years, while it was 69.1 years for the FTRCT group. Additionally, sarcopenia was not significantly associated with RCT severity (*p* = 0.612), as detailed in Table 4.

### 3.4. Multivariable Logistic Regression Analysis: Factors Associated with Sarcopenia and RCT

Ultimately, a multivariable logistic regression analysis was conducted to determine the factors associated with RCTs. The analysis revealed that chronic kidney injury was significantly linked to RCTs, with an OR of 7.845 and a 95% CIs of 2.099–51.113 (*p* = 0.008). Additionally, calcium levels showed a significant association with RCTs, with an OR of 1.273 and a 95% CIs of 1.019–1.598 (*p* = 0.035), as presented in Table 5. This indicates that higher calcium levels are associated with an increased risk of RCTs, and patients with chronic kidney injury have a higher likelihood of experiencing RCTs.

## 4. Discussion

We conducted a retrospective study to form sarcopenia and RCT groups based on body composition tests and MRI results. The RCT group was further categorized by the size and severity of the tears. Through multivariate logistic regression analysis, we could not find the association between sarcopenia and the development of RCT. Additionally, neither the size nor the severity of RCTs was correlated with sarcopenia.

Sarcopenia and RCTs may be connected through physiological changes—such as reductions in muscle mass, strength, and neuromuscular function—as well as anatomical changes involving degenerative modifications in muscle and tendon structures. Sarcopenia is a gradual, non-pathological phenomenon related to aging and is marked by a decline in both skeletal muscle mass and functional capabilities [23]. This decline in muscle mass associated with aging results in a decrease in muscle strength, particularly beginning in the elderly stage of life, with a significant worsening occurring thereafter [24,25]. This condition is linked to age-related declines in hormone levels, reduced caloric intake, a sedentary lifestyle, and metabolic disorders such as diabetes mellitus [9,25,26]. In relation to RCTs, Gibbons et al. showed that the rotator cuff muscles experience an active cycle of degeneration and regeneration, and that increased inflammation can worsen the degenerative process associated with progressive rotator cuff disease. While RCT itself is a localized condition, certain systemic diseases—such as diabetes mellitus, hypercholesterolemia, thyroid disorders, and osteoporosis—are recognized as being linked to the pathophysiology of RCTs [27].

Multiple studies examining the relationship between sarcopenia and RCTs have yielded varying results. Chung et al. investigated the potential link between sarcopenia and RCTs using bioelectrical impedance analysis for body composition assessment, which is considered less reliable than DEXA scans [6]. Their study compared two groups, consisting of 48 patients with RCT and 48 controls without RCTs, and concluded that sarcopenia was more pronounced in patients with chronic symptomatic FTRCT. Conversely, Atala et al. found that the prevalence of sarcopenia in RCT patients was similar to that in an age- and sex-matched control group, suggesting that sarcopenia is not an independent risk factor for RCT [15]. Han et al. utilized ultrasound for diagnosing RCTs in community-dwelling older adults and observed that individuals with sarcopenia had a higher risk of shoulder pain and exhibited consistent tendinopathic changes in the supraspinatus tendon. However, they noted that sarcopenia was less likely to be associated with severe rotator cuff pathologies, such as tendon tears [28].

In contrast to prior studies, we analyzed a larger sample size, with 144 participants in both the RCT and non-RCT groups after PSM. We also evaluated the size and severity of the tears, using body composition tests and DEXA, alongside baseline data that included past medical histories and blood tests. To maintain demographic consistency, we matched 144 individuals based on age, sex, and BMD. Our findings indicated that only calcium levels and chronic kidney injury were significantly associated with RCTs.

These divergent results prompt consideration of additional factors. One study indicated a positive correlation between higher BMI and an increased incidence of RCTs [29]. However, in our analysis, the BMI was lower in the sarcopenia group, implying that it may not directly correlate with the occurrence of RCTs (Table 1). Furthermore, previous studies did not adjust for BMD in their analyses, despite evidence suggesting that lower BMD could affect both the onset and recovery from an RCT [30,31].

The lack of a relationship between sarcopenia and RCT may arise from the differing ages at which these conditions onset, even though both are degenerative diseases whose prevalence increases with age. According to earlier research, sarcopenia becomes notably prevalent among older adults aged 65 and above, while RCTs can be identified in patients as early as in their 50s [32,33,34]. Population-based studies have shown average ages of 59 for small tears, 62 for medium tears, 64 for large tears, and 66 for massive tears [35]. Additionally, the development of an RCT is associated with higher physical activity levels; thus, individuals in their 50s may be more likely to experience a symptomatic RCT due to their greater engagement in physical and sporting activities compared to those over 65.

This study has several limitations. First, its cross-sectional design makes it impossible to establish definitive cause-and-effect relationships, highlighting the need for prospective studies to clarify origins and outcomes. Second, while our study focused on the body composition aspect of sarcopenia, the definitions of sarcopenia can vary, often including considerations of muscle strength, function, and muscle mass index. Although it would be beneficial to examine these criteria collectively, data collection was restricted by the retrospective nature of this study. Third, the control group lacked MRI evaluations. Although this group was composed of individuals without shoulder symptoms or a surgical history, it is important to recognize the potential presence of asymptomatic RCTs, particularly since the incidence of RCTs tends to increase with age, with many cases possibly remaining asymptomatic.

## 5. Conclusions

In conclusion, while sarcopenia and RCTs are both prevalent conditions among aging populations, our study did not find a significant relationship between sarcopenia and RCT occurrence, size, or severity. Instead, calcium levels and chronic kidney injury emerged as significant predictors of RCTs. These results suggest that the relationship between sarcopenia and RCTs may be more complex than previously understood, emphasizing the need for further prospective research to clarify the underlying mechanisms and clinical implications of these findings. Also, clinicians should bear in mind that a patient may still have the possibility of an RCT, even though the patient is not sarcopenic.

## Figures and Tables

**Table 1:** Differences between the sarcopenia and non-sarcopenia groups before PSM.

Variable	Non-Sarcopenia (n = 292)	Sarcopenia (n = 176)	*p*-Value
Age (years)	66.8 ± 6.1	68.6 ± 6.9	0.003 *
Sex, n (%)			0.012 ^†^
Male	88 (30.1)	73 (41.5)	
Female	292 (69.9)	103 (58.5)	
Height (m)	1.6 ± 0.08	1.6 ± 0.09	0.233 *
Weight (kg)	62.5 ± 9.2	57.9 ± 9.7	<0.001 *
BMI (kg/m^2^)	25.0 ± 3.1	22.8 ± 3.2	<0.001 *
BMD (T-score)	−0.9 ± 1.3	−1.3 ± 1.3	0.004 *
BMD, n (%)			<0.001 ^†^
Normal	74 (25.3)	31 (17.6)	
Osteopenia	139 (47.6)	72 (40.9)	
Osteoporosis	79 (27.1)	73 (41.5)	
RCT, n (%)			0.441 ^†^
Yes	120 (41.1)	66 (37.5)	
No	172 (58.9)	110 (62.5)	
Laboratory parameters			
Calcium (mg/dL)	9.31 ± 0.51	9.12 ± 0.6	0.001 *
Phosphorus (mg/dL)	3.51 ± 0.6	3.43 ± 0.7	0.197 *
Vitamin D (ng/mL)	21.6 ± 9.8	21.0 ± 11.7	0.639 *
Comorbidity, n (%)			
Hypertension			0.136 ^†^
Yes	130 (44.5)	66 (37.5)	
No	162 (55.5)	110 (62.5)	
Diabetes mellitus			0.372 ^†^
Yes	61 (20.9)	43 (24.4)	
No	231 (79.1)	133 (75.6)	
Chronic kidney injury			<0.001 ^†^
Yes	18 (6.2)	28 (15.9)	
No	274 (93.8)	148 (84.1)	
Osteoporotic fracture of the spine or hip			0.362 ^†^
Yes	53 (18.2)	38 (21.6)	
No	239 (81.8)	138 (78.4)	

* *t-*test; ^†^ Pearson’s chi-square test; RCT—rotator cuff tear; BMI—body mass index; BMD—bone mineral density.

**Table 2:** Univariate analysis of factors associated with RCTs.

Variable	Non-RCT (n = 144)	RCT (n = 144)	*p*-Value
Age (years)	68.0 ± 5.3	68.0 ± 7.3	0.985 *
Sex, n (%)			>0.99 ^†^
Male	53 (36.8)	54 (37.5)	
Female	91 (63.2)	90 (62.5)	
Height (m)	1.6 ± 0.1	1.6 ± 0.1	0.606 *
Weight (kg)	61.3 ± 9.4	60.7 ± 10.4	0.633 *
BMI (kg/m^2^)	24.4 ± 3.3	24.0 ± 3.5	0.297 *
BMD (T-score)	−1.5 ± 1.1	−1.5 ± 1.2	0.835 *
BMD, n (%)			0.938 ^†^
Normal	41 (28.5)	43 (29.9)	
Osteopenia	72 (50.0)	69 (47.9)	
Osteoporosis	31 (21.5)	32 (22.2)	
Body status, n (%)			0.719 ^†^
Non-sarcopenia	83 (57.6)	87 (60.4)	
Sarcopenia	61 (42.4)	57 (39.6)	
Laboratory parameters			
Calcium (mg/dL)	9.17 ± 0.6	9.31 ± 0.5	0.028 *
Phosphorus (mg/dL)	3.47 ± 0.7	3.55 ± 0.6	0.305 *
Vitamin D (ng/mL)	23.3 ± 10.3	20.1 ± 10.7	0.055 *
Comorbidity, n (%)			
Hypertension			0.230 ^†^
Yes	64 (44.4)	53 (36.8)	
No	80 (55.6)	91 (63.2)	
Diabetes mellitus			>0.99 ^†^
Yes	29 (20.1)	30 (20.8)	
No	115 (79.9)	114 (79.2)	
Chronic kidney injury			0.005 ^†^
Yes	2 (1.4)	14 (9.7)	
No	142 (98.6)	130 (90.3)	
Osteoporotic fracture of the spine or hip			>0.99 ^†^
Yes	28 (19.4)	29 (20.1)	
No	116 (80.6)	115 (79.9)	

* *t*-test; ^†^ Pearson’s chi-squared test; RCT—rotator cuff tear; BMI—body mass index; BMD—bone mineral density.

**Table 3:** Comparison of factors associated with RCT size.

Variable	Small (n = 66)	Medium (n = 34)	Large (n = 24)	Massive (n = 20)	*p*-Value
Age (years)	67.3 ± 8.3	66.2 ± 6.7	71.1 ± 5.1	69.8 ± 6.1	0.041 *
Sex, n (%)					0.049
Male	20 (30.3)	18 (52.9)	6 (25.0)	10 (50.0)	
Female	46 (69.7)	16 (47.1)	18 (75.0)	10 (50.0)	
Height (m)	1.6 ± 0.1	1.6 ± 0.1	1.6 ± 0.1	1.6 ± 0.1	0.104 *
Weight (kg)	60.0 ± 10.9	60.6 ± 10.7	61.2 ± 9.3	62.8 ± 9.9	0.763 *
BMI (kg/m^2^)	23.9 ± 3.7	23.3 ± 3.5	25.2 ± 3.5	24.2 ± 2.5	0.227 *
BMD (T-score)	−1.5 ± 1.3	−1.5 ± 1.3	−1.9 ± 1.1	−1.1 ± 1.0	0.179 *
BMD, n (%)					0.672
Normal	19 (28.8)	11 (32.4)	5 (20.8)	8 (40.0)	
Osteopenia	33 (50.0)	14 (41.2)	12 (50.0)	10 (50.0)	
Osteoporosis	14 (21.2)	9 (26.5)	7 (29.2)	2 (10.0)	
Body status, n (%)					0.183
Non-sarcopenia	34 (51.5)	25 (73.5)	15 (62.5)	13 (65.0)	
Sarcopenia	32 (48.5)	9 (26.5)	9 (37.5)	7 (35.0)	
Laboratory parameters					
Calcium (mg/dL)	9.22 ± 0.6	9.41 ± 0.3	9.37 ± 0.4	9.28 ± 0.5	0.412 *
Phosphorus (mg/dL)	3.58 ± 0.6	3.43 ± 0.6	3.62 ± 0.6	3.33 ± 0.6	0.247 *
Vitamin D (ng/mL)	19.1 ± 11.5	20.0 ± 7.7	21.3 ± 9.3	21.6 ± 13.4	0.792 *
Comorbidity, n (%)					
Hypertension					0.590
Yes	24 (36.4)	11 (32.4)	8 (33.3)	10 (50.0)	
No	42 (63.6)	23 (67.6)	16 (66.7)	10 (50.0)	
Diabetes mellitus					0.726
Yes	15 (22.7)	8 (23.5)	3 (12.5)	4 (20.0)	
No	51 (77.3)	26 (76.5)	21 (87.5)	16 (80.0)	
Chronic kidney injury					0.288
Yes	6 (9.1)	1 (2.9)	4 (16.7)	3 (15.0)	
No	60 (90.9)	33 (97.1)	20 (83.3)	17 (85.0)	
Osteoporotic fracture of the spine or hip					0.374
Yes	17 (25.8)	4 (11.8)	5 (20.8)	3 (15.0)	
No	49 (74.2)	30 (88.2)	19 (79.2)	17 (85.0)	

* One-way ANOVA test; RCT, rotator cuff tear; BMI, body mass index; BMD, bone mineral density.

**Table 4:** Comparison of factors associated with RCT severity.

Variable	PTRCT (n = 40)	FTRCT (n = 104)	*p*-Value
Age (years)	65.0 ± 7.2	69.1 ± 7.1	0.002 *
Sex, n (%)			0.337 ^†^
Male	12 (30.0)	42 (40.4)	
Female	28 (70.0)	62 (59.6)	
Height (m)	1.6 ± 0.1	1.6 ± 0.1	0.558 *
Weight (kg)	62.7 ± 10.4	60.0 ± 10.4	0.156 *
BMI (kg/m^2^)	24.6 ± 3.6	23.8 ± 3.4	0.187 *
BMD (T-score)	−1.5 ± 1.1	−1.5 ± 1.3	0.764 *
BMD, n (%)			0.358 ^†^
Normal	10 (25.0)	33 (31.7)	
Osteopenia	23 (57.5)	46 (44.2)	
Osteoporosis	7 (17.5)	25 (24.0)	
Body status, n (%)			0.612 ^†^
Non-sarcopenia	26 (65.0)	61 (58.7)	
Sarcopenia	14 (35.0)	43 (41.3)	
Laboratory parameters			
Calcium (mg/dL)	9.32 ± 0.4	9.28 ± 0.5	0.868 *
Phosphorus (mg/dL)	3.63 ± 0.5	3.47 ± 0.6	0.540 *
Vitamin D (ng/mL)	20.0 ± 9.7	20.2 ± 11.1	0.917 *
Comorbidity, n (%)			
Hypertension			0.214 ^†^
Yes	11 (27.5)	42 (40.4)	
No	29 (72.5)	62 (59.6)	
Diabetes mellitus			0.593 ^†^
Yes	10 (25.0)	20 (19.2)	
No	30 (75.0)	84 (80.8)	
Chronic kidney injury			0.134 ^†^
Yes	1 (2.5)	13 (12.5)	
No	39 (97.5)	91 (87.5)	
Osteoporotic fractures of the spine or hip			0.503 ^†^
Yes	10 (25.0)	19 (18.3)	
No	30 (75.0)	85 (81.7)	

* *t*-test; ^†^ Pearson’s chi-squared test; RCT—rotator cuff tear; BMI—body mass index; BMD—bone mineral density.

**Table 5:** Multivariable logistic regression analysis of factors associated with RCTs.

Variable	OR (95% CI)	*p*-Value
Laboratory parameters		
Calcium (mg/dL)	1.273 (1.019–1.598)	0.035
Comorbidity, n (%)		
Chronic kidney injury	7.845 (2.099–51.113)	0.008

RCT—rotator cuff tear; OR—odds ratio; CI—confidence interval.

## Data Availability

The datasets generated and/or analysed during the current study are not publicly available but are available from the corresponding author on reasonable request.

## Supplementary Materials

The following supporting information can be downloaded at https://www.mdpi.com/article/10.3390/jcm14010220/s1, Figure S1: Standardized mean differences of the covariates after propensity score matching; Table S1: Univariate analysis of factors associated with RCT in patients aged 60–70 years before propensity score matching (PSM).
