# Supplementary material for: Examining the Relationship Between Sarcopenia and Rotator Cuff Tears: A Retrospective Comparative Study
Source: J Clin Med. 2025 Jan 2;14(1):220. doi: 10.3390/jcm14010220 (PMC11721911; doi:10.3390/jcm14010220)
Supplement: Supplementary file 1 [file jcm-14-00220-s001.zip › Supplemental Table S1.pdf]

Supplemental Table S1. Univariate analysis of factors associated with RCT in patients aged 60-70 years before propensity score matching (PSM).

**Variable**	**Non-RCT (n = 180)**	**RCT (n = 91)**	***p*-Value**
**Age (years)**	64.9 ± 3.3	65.5 ± 3.0	0.127*
**Sex, n (%)**			0.001†
Male	45 (25.0)	45 (49.5)	
Female	135 (75.0)	46 (50.5)	
**Height (m)**	1.6 ± 0.1	1.6 ± 0.1	0.005*
**Weight (kg)**	60.7 ± 9.4	62.7 ± 9.9	0.596*
**BMI (kg/m²)**	24.3 ± 3.4	24.1 ± 3.1	0.079*
**BMD (T-score)**	-1.5 ± 1.4	-0.9 ± 1.4	0.288*
**BMD, n (%)**			0.055†
Normal	31 (17.2)	27 (29.7)	
Osteopenia	94 (52.2)	38 (41.8)	
Osteoporosis	55 (30.6)	26 (28.5)	
**Body status, n (%)**			0.335†
Non-sarcopenia	114 (63.3)	63 (69.2)	
Sarcopenia	66 (36.7)	28 (30.8)	
**Laboratory parameters**			
Calcium (mg/dL)	9.2 ± 0.5	9.4 ± 0.4	0.087*
Phosphorus (mg/dL)	3.5 ± 0.6	3.5 ± 0.6	0.892*
Vitamin D (ng/mL)	23.4 ± 10.5	21.2 ± 10.0	0.311*
**Comorbidity, n (%)**			
Hypertension			0.646†
Yes	75 (41.7)	35 (38.5)	
No	105 (58.3)	56 (61.5)	
Diabetes mellitus			0.164†
Yes	36 (20.0)	25 (27.5)	
No	144 (80.0)	66 (72.5)	
Chronic kidney injury			0.111†
Yes	18 (10.0)	4 (4.4)	
No	162 (90.0)	87 (95.6)	
Osteoporotic fracture of the spine or hip			0.766†
Yes	31 (17.2)	17 (18.7)	
No	149 (82.8)	74 (81.3)	

* T-test; † Pearson's chi-square test; RCT, rotator cuff tear; BMI, body mass index; BMD, bone mineral density
