# Supplementary figures and images for: Examining the Relationship Between Sarcopenia and Rotator Cuff Tears: A Retrospective Comparative Study
Source: J Clin Med. 2025 Jan 2;14(1):220. doi: 10.3390/jcm14010220 (PMC11721911; doi:10.3390/jcm14010220)

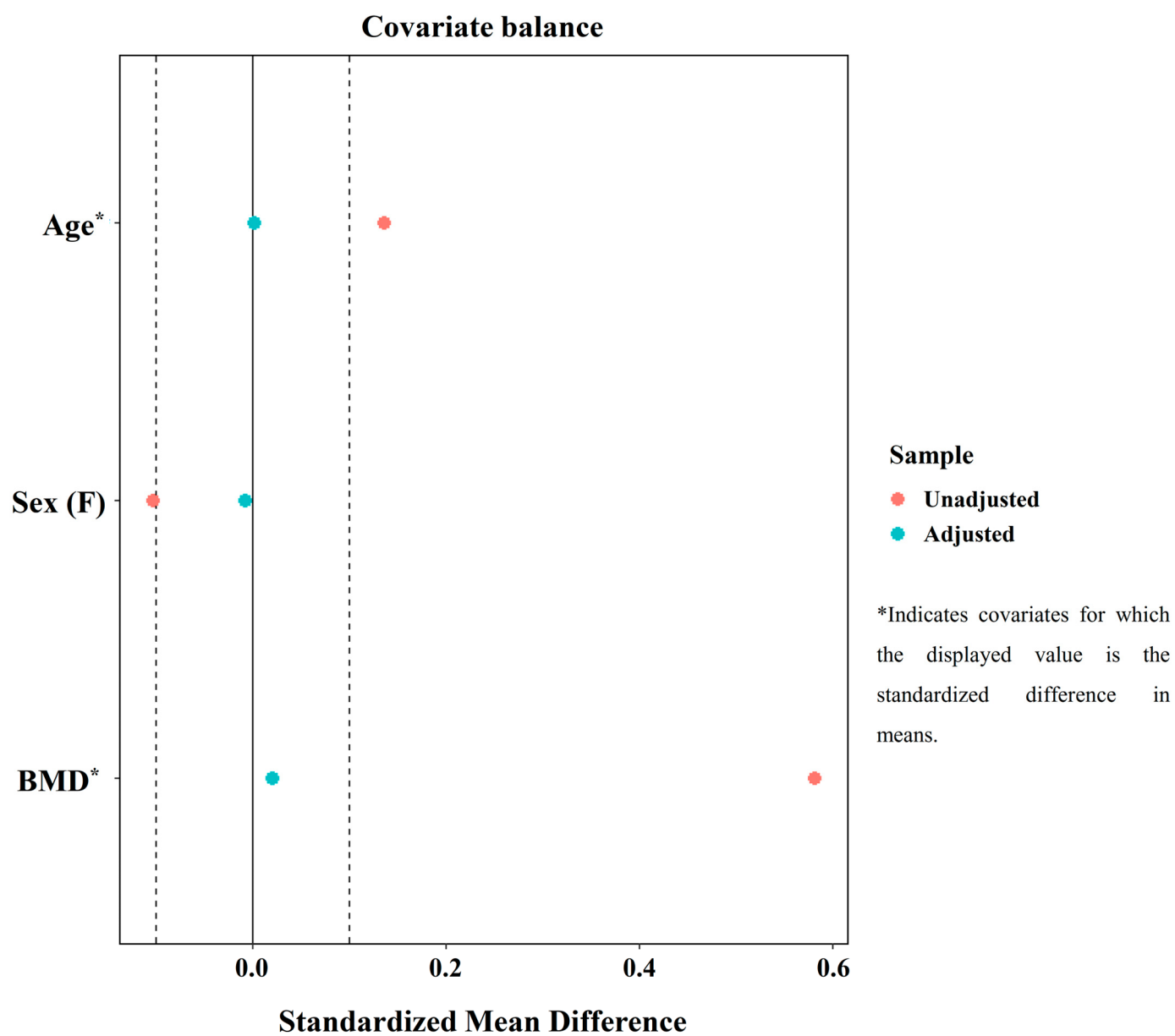

**Figure S1:** Standardized mean differences of the covariates after propensity score matching.

Supplement: Supplementary file 1 [file jcm-14-00220-s001.zip › Supplementary Figure S1.pdf]
